# Complex Effects of Sertraline and Citalopram on In Vitro Murine Breast Cancer Proliferation and on In Vivo Progression and Anxiety Level

**DOI:** 10.3390/ijms23052711

**Published:** 2022-02-28

**Authors:** Michal Taler, Irit Gil-Ad, Iris Brener, Shay Henry Hornfeld, Abraham Weizman

**Affiliations:** 1 Laboratory of Biological and Molecular Psychiatry, Felsenstein Medical Research Center, 39 Jabotinski St., Petah Tikva 4941492, Israel; michalt@tauex.tau.ac.il (M.T.); iritgilad10@gmail.com (I.G.-A.); iris.brener@gmail.com (I.B.); hsh1980@walla.co.il (S.H.H.); 2 Petah Tikva and Department of Psychiatry, Sackler Faculty of Medicine, Tel Aviv University, 35 Klachkin St., Tel Aviv 6997801, Israel; 3 Research Unit, Geha Mental Health Center, 1 Helsinki St., Petah Tikva 4910002, Israel

**Keywords:** antidepressants, SSRIs, breast cancer, stress, tumor growth, oncological pharmacology

## Abstract

Some selective serotonin reuptake inhibitors (SSRIs), primarily sertraline, demonstrate anti-proliferative activity in malignant cell-lines and in xenografted mouse models of colorectal tumor. There is, however, a paucity of comparative studies on the anti-tumor effects of SSRIs. We compared the in vitro and in vivo effects of sertraline and citalopram on murine 4T1 breast cancer. Grafted mice were used to determine the rate of tumor growth and survival as well as the impact of stress and antidepressant treatment on tumor progression and mortality and on pro-inflammatory cytokines. Sertraline, in the micromolar range, but not citalopram, induced a significant in vitro concentration-dependent inhibition of murine 4T1 cell proliferation and splenocyte viability. In contrast, sertraline (10 mg/kg/d), enhanced in vivo tumor growth. Contrary to the study’s hypothesis, chronic mild stress did not modify tumor growth in grafted mice. The in vitro effects of sertraline on tumor growth seem to be the opposite of its in vivo effects. The impact of sertraline treatment on humans with breast cancer should be further investigated.

## 1. Introduction

Breast cancer is the most common malignancy among women worldwide and comprises 18% of all female cancers [1]. The diagnosis of breast cancer and the following period of therapy are stressful times for the patient. Often antidepressants, mainly serotonin reuptake inhibitors (SSRIs), are prescribed to ameliorate the resulting anxiety and depression [2,3,4]. 

The current authors provided, in previous studies, evidence of the anti-proliferative activity mediated by the apoptotic effect of some SSRIs, particularly in vitro sertraline and paroxetine (IC_50_ = 8–15 µM) in several cancer cell-lines, including human colorectal carcinoma HT29 [5], Jurkat Lymphoma [6] and human glioblastoma U87 [7]. 

Similar effects were also observed by the authors with regard to immune cells. In vitro investigation showed that the SSRIs paroxetine and sertraline suppress cell viability and responsiveness to mitogen-induced proliferation of human T lymphocytes and rat splenocytes. This effect was achieved by apoptosis induced by cell cycle arrest and accompanied by a decrease in the secretion of pro-inflammatory cytokines, including tumor necrosis factor (TNF)-α [8]. 

Meanwhile, reports by other authors showed that, depending on the degree of lymphocyte activation, the SSRI fluoxetine may affect T cell proliferation in a bidirectional manner. At mitogenic concentrations (2 µg/mL) of concavalin A (Con A), fluoxetine exerted an inhibitory effect on cellular proliferation, whereas at submitogenic Con A concentrations (1 µg/mL), fluoxetine stimulated the cellular proliferation [9]. 

The authors of the current study furthermore reported that sertraline and paroxetine at low concentrations (1 µM), increase in vitro human neuroblastoma SHSY5Y cell viability, while citalopram, fluoxetine, reboxetine and mirtazapine at the same concentration does not. The anti-proliferative activity of sertraline, however, was observed only at higher concentrations [10]. These findings indicate a potential concentration-dependent effect of sertraline and paroxetine related to tumor cell proliferation. The apoptotic effect of fluoxetine was observed in the human gastric cancer cell line AGS [11]. Moreover, combining SSRIs with anticancer agents was reported to augment the efficacy of some of these agents, [12] and both fluoxetine and paroxetine were suggested as promising adjuvants for patients with hepatocellular carcinoma, non-small cell lung cancer [13], and breast cancer [14].

Additionally, stress-associated physiological changes, like activation of the hypothalamic-pituitary-adrenal (HPA) axis, were shown to accelerate the progression of some cancer types. Studies in animals and in humans have demonstrated that stress-related molecular parameters released during chronic HPA axis activation and depression may suppress the immune response, leading to aggravation of the tumor progression [15]. Notably, SSRIs could be effective in normalizing the hyperactive HPA system [16]. When prescribing antidepressants for oncological patients, however, a balance should be found between their effectiveness, safety, and possible interference with the efficacy of the anti-cancer drugs [17]. Li et al. have shown that sertraline relieves depression in patients with cancer and improves their quality of life [18]. In another study, however, high doses of sertraline did not improve mood symptoms, wellbeing, or survival of patients with advanced-stage cancer but without major depression [19]. Notably, several epidemiological studies have shown that chronic use of SSRIs can lower the risk of developing some types of cancer [20,21,22,23]. 

The present study evaluated the following:

- the comparative in vitro anti-proliferative effect of sertraline and citalopram on the murine breast cancer 4T1 cell-line, growth and survival,
- the in vivo interaction among SSRIs, stress and tumor progression in a mouse 4T1 breast cancer grafted model and ex-vivo splenocyte proliferation, and
- secretion of the pro-inflammatory cytokine TNF-α, in response to LPS stimulation, which may affect cancer progression.

In this series of experiments we compared the anti-proliferative effects of sertraline, which, as mentioned above, we have shown to possess anti-proliferative properties and citalopram, which we have shown not to possess such properties [10]. We attempted to determine the possible complex relations between pro-inflammatory cytokine production, stress, major depression, and tumor growth, as well as the potential role and effectiveness of specific antidepressant interventions in cellular and in vivo models of cancer. The ex vivo studies of TNF-α responses to LPS stimulation of cultured splenocytes further highlight this complexity. 

## 2. Results

### 2.1. In Vitro—4T1 Cell-Line and Splenocytes

**Figure 1:**
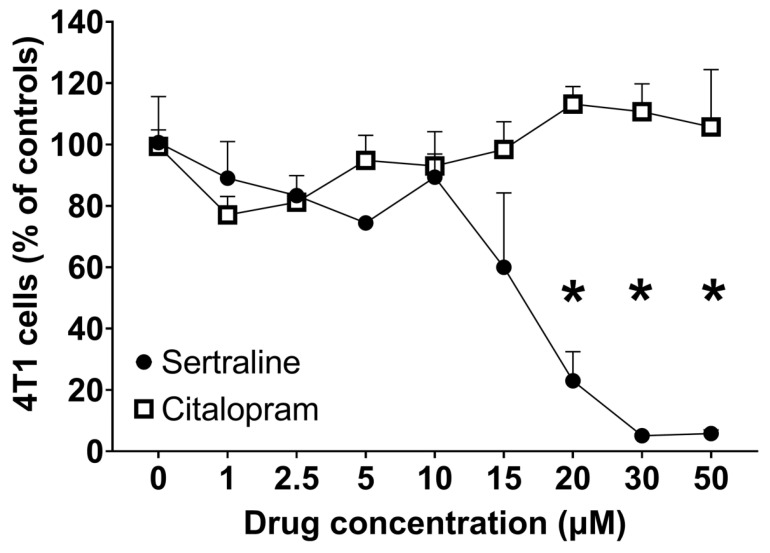
Effect of various concentrations of SSRIs on 4T1 cell viability—Points represent Mean ± SE of 3 separate experiments, expressed as % of controls. Viability was assessed by Neutral Red following 24 h incubation under normal conditions (no starvation). * *p* < 0.05 vs. basal.

The effects of various concentrations of sertraline and citalopram on the viability of the 4T1 murine mammary carcinoma cell-line, as determined by the Neutral Red method were studied for 24 h under normal conditions (no starvation). Sertraline induced an up to 90% concentration-dependent decrease in cell viability with significant effects at 20–50 µM, while none of the citalopram doses modified cell viability significantly. RM-ANOVA showed a significant difference (F_(8, 32)_ = 10.36, *p* < 0.01) between the impact of the two SSRI agents (sertraline and citalopram) on cell viability (Figure 1).

The same procedure was carried out in serum deficient conditions (starvation condition) and neither sertraline nor citalopram were found to significantly affect cell viability (data not shown).

**Figure 2:**
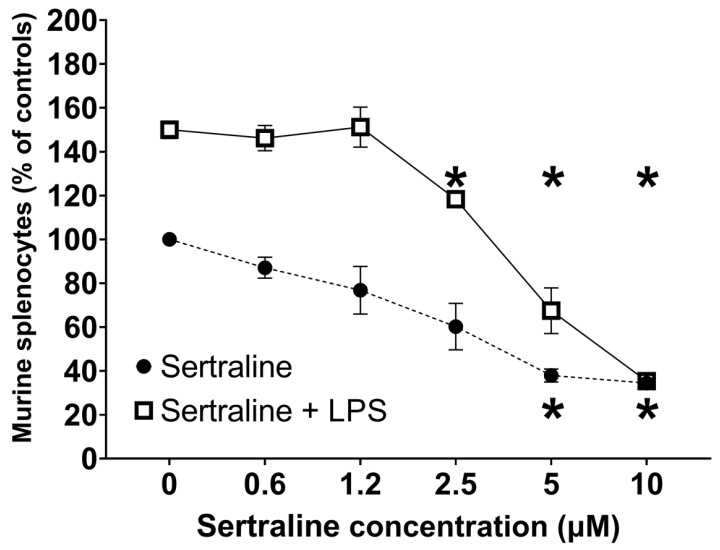
Effect of sertraline on viability of murine splenocytes—Viability was measured under basal conditions and following exposure to LPS (0.1 µg/well). Points represent mean ± SE of three separate experiments, expressed as percentage of control. Proliferation was assessed by Alamar Blue following 48 h incubation. RM-ANOVA revealed *p* < 0.001. * *p* < 0.05 vs. basal.

Under normal conditions, RM-ANOVA shows that LPS (0.1 µg/well) increased cell viability (F_(1,4)_ = 131.86, *p* < 0.001) by 60% as compared to sertraline alone. Sertraline caused significant concentration-dependent decreases (F_(5,20)_ = 64.77, *p* < 0.001) in splenocyte viability with and without LPS exposure. Post-hoc multiple comparisons show that sertraline alone induced a concentration-dependent suppression in splenocyte viability that reached significance at concentrations of 5–10 µM (*p* < 0.05). Under LPS exposure, sertraline induced a steep decline in splenocyte viability (30–90%) starting at 2.5 µM (Figure 2). Contrary to sertraline, citalopram did not modify splenocyte viability either alone or under LPS exposure. 

### 2.2. In Vivo—CMS

**Figure 3:**
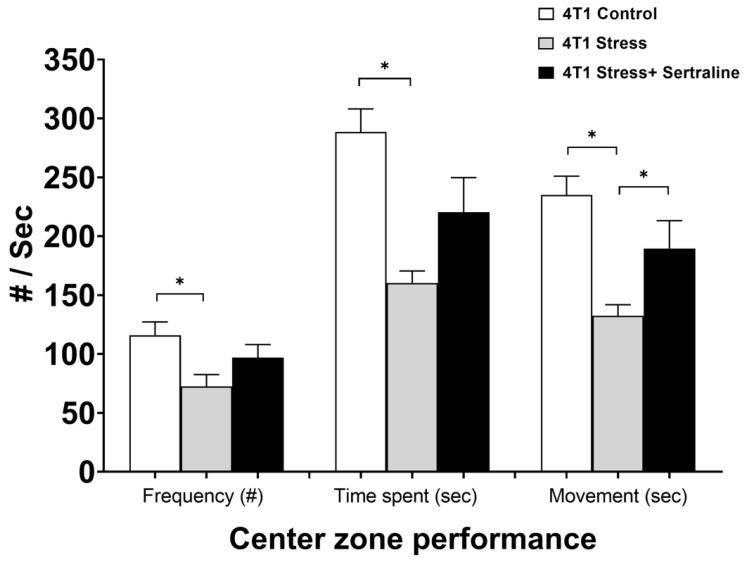
Behavior of 4T1cell inoculated mice in Open Field test—Effects of chronic mild stress (CMS) and sertraline treatment (5 mg/kg/d, seven days after 4T1 cell inoculation) on center zone performance in Open Field test were measured by frequency of entry into center zone, time spent in it and duration of movement in the center. Results are expressed as mean ± SE of three separate experiments, which included five mice per group. * *p* < 0.05.

**Figure 4:**
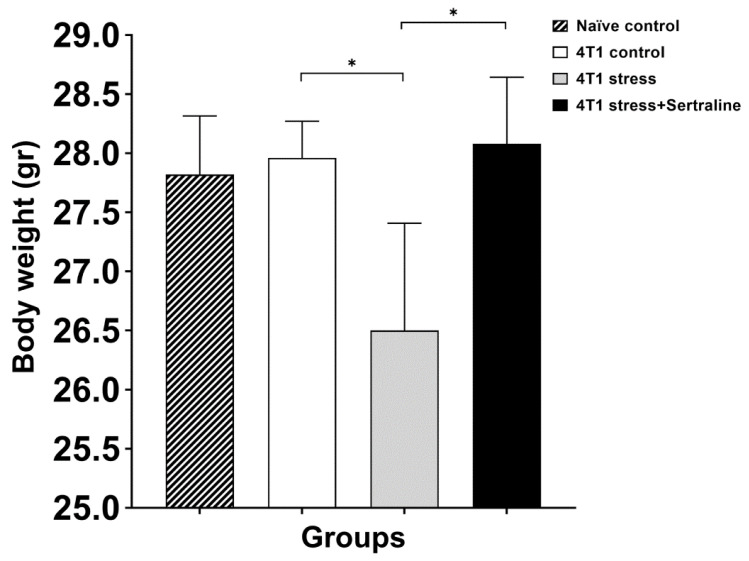
Effects of the various treatments on body weight—Each bar represents mean ± SE body weight of the various groups after six weeks of CMS with or without sertraline treatment (5 mg/kg/d). * *p* < 0.05.

Figure 3 shows the effect of CMS and sertraline treatment on anxiety parameters as demonstrated in the Open Field test by center zone activity, namely, frequency of entry into the center zone, time spent in the center zone and duration of movement (being in motion) in the center zone. Decreased activity in the center of the field, when measured seven days after 4T1 cell inoculation, indicates that CMS significantly increased the level of anxiety in the mice. Sertraline treatment (5 mg/kg/d) prevented this effect (F_(2, 16)_ = 4.05, *p* < 0.05). Moreover, stressed 4T1 inoculated mice showed significantly (*p* < 0.05) lower body weight after 31 days of CMS as compared to 4T1 unstressed controls. Sertraline treatment reversed this effect (Figure 4). However, analysis of tumor weight at autopsy did not reveal differences between the three groups of tumor bearing mice.

**Figure 5:**
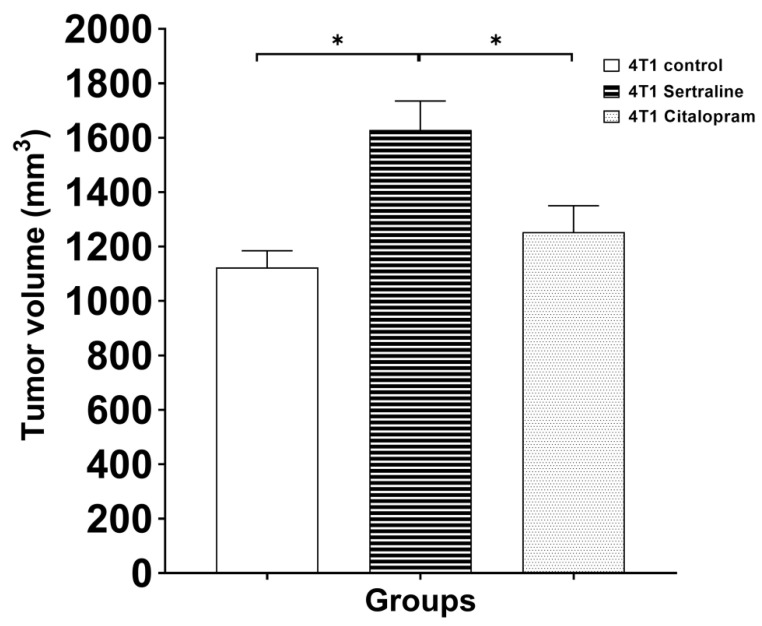
Effect of sertraline or citalopram (10 mg/kg/d) on tumor volume of 4T1 cell inoculated mice—Tumor volume in mice treated with SSRIs (10 mg/kg/d) 20 days after 4T1 cell inoculation are expressed as mean± SE (*n* = 5 in each group). * *p* < 0.05.

When tumor volume was determined on day 20 after cell inoculation, ANOVA showed significant differences among the groups (F_(2, 27)_ = 8.26, *p* < 0.01). Post-hoc analysis revealed that both 4T1 controls and 4T1 citalopram-treated mice had significantly lower tumor volume than 4T1 sertraline-treated mice (*p* < 0.01). 4T1 controls and 4T1 citalopram- treated mice did not show significant differences in tumor volumes (Figure 5), and neither were there differences on day 32 at autopsy (F_(2, 12)_ = 2.77, *p* = 0.10). 

The body weight of Balb/c mice were not significantly affected by the two SSRIs (sertraline and citalopram 10 mg/kg/d; F_(3, 33)_ = 2.518, *p* = 0.075).

**Figure 6:**
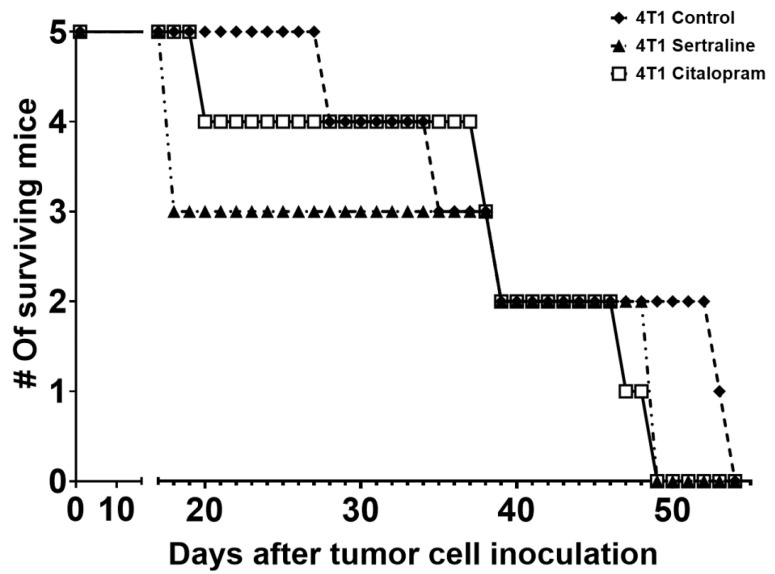
Survival of 4T1 cell grafted mice—Mice were treated with sertraline or citalopram (10 mg/kg/d each) and monitored up to day 52 after grafting, when the last mouse died due to tumor mass formation. Each group consisted of five mice.

Next, the survival rates of 4T1 inoculated mice (five per group) that were kept undisturbed until death occurred were compared to 4T1 controls. Although no significant differences were found between the groups, it should be noted that the SSRI-treated, 4T1 grafted mice died earlier than the untreated mice (Figure 6).

### 2.3. Ex Vivo—Splenocyte Proliferation 

**Figure 7:**
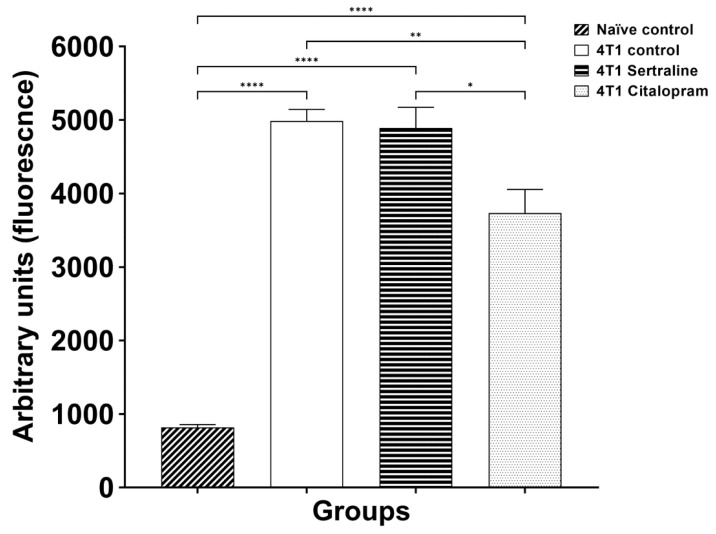
Ex Vivo proliferation of splenocytes derived from the four groups—Comparison of viability of splenocytes derived from 4T1 cell grafted groups, treated with SSRIs (10 mg/kg/d) vs. 4T1 controls and naïve mice. Proliferation was measured following 48 h incubation with LPS (0.1 µg/well) by Alamar Blue fluorescence. Each bar represents mean ± SE of three separate experiments (*n* = five in each group). * *p* < 0.05; ** *p* < 0.01, **** *p* < 0.001.

This analysis was performed in spleens extracted from mice described in Experiment 2 and sacrificed on day 32. Splenocytes from the four groups were cultured and exposed to LPS (0.1 µg/well) and significant differences were demonstrated between the groups (F_(3, 15)_ = 57.97, *p* < 0.01; Figure 7). In post-hoc analysis, the response of the 4T1 citalopram splenocyte proliferation was significantly lower than that of the 4T1 control and the 4T1 sertraline groups (*p* < 0.01). No such differences were found with sertraline.

**Figure 8:**
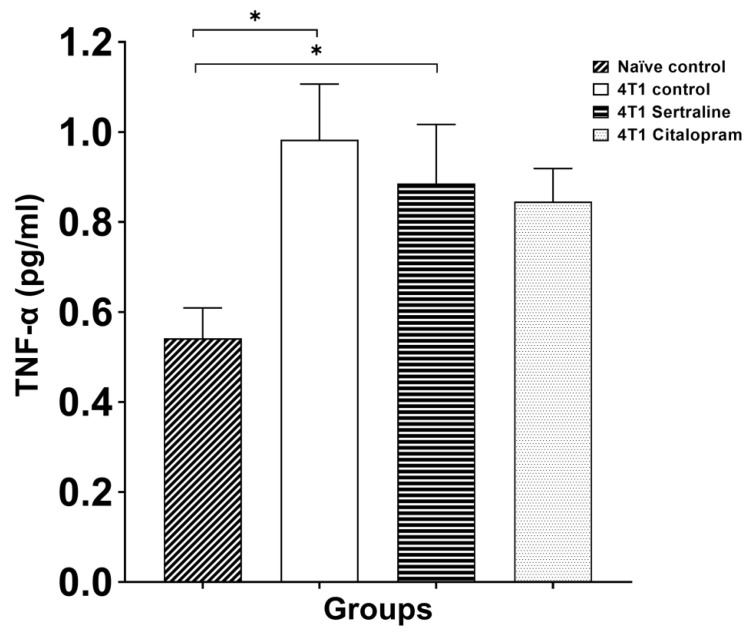
Ex Vivo effects of SSRIs on TNF-α secretion of splenocytes—Splenocytes were derived from the four groups. Splenocytes of SSRI treated 4T1 mice (10 mg/kg/d) were compared to those of 4T1 controls and naïve mice. Ex-vivo TNF-α secretion from splenocytes was measured following LPS stimulation. Each bar represents mean ± SE of three separate experiments (*n* = five in each group), expressed as % of controls, as assessed by ELISA following 48 h of incubation. ANOVA revealed *p* < 0.01 between groups. * *p* < 0.05.

Figure 8 shows the ex-vivo secretion of TNF-α from the cultured splenocytes in response to LPS stimulation. ANOVA demonstrated a tendency toward significant differences between the groups (F_(3, 15)_ = 2.866, *p* = 0.072). A t-test comparison to splenocytes derived from naïve mice found significantly higher TNF-α levels in the 4T1 controls and in the 4T1 sertraline group (*p* < 0.05), but not in the 4T1 citalopram treated splenocytes.

## 3. Discussion

This study provides evidence that the administration of 10 mg/kg/d of the antidepressant sertraline, but not citalopram, to breast cancer inoculated female mice may accelerate tumor growth and cancer progression. Clinical studies indicate that stress, chronic depression, and other psychological factors known to have suppressive effects on the immune system may influence cancer onset and progression [15,16]. 

Antidepressants are widely used to treat depression and anxiety in cancer patients [2,3]. In addition to the anti-depressive activity, SSRIs were also found to have immune modulating and anti-proliferative effects [5,6,7,8,9]. In previous studies the authors demonstrated differential effects of antidepressants on several cell-lines including colon carcinoma, glioma, neuroblastoma and lymphoma. In those studies sertraline and paroxetine exhibited antiproliferative and proapoptotic activity while fluoxetine, reboxetine and clomipramine showed small or no effects [6,8,24]. The current study used sertraline and citalopram, which are considered safe and are commonly prescribed to cancer patients [3].

Sertraline was also found to have some in vivo anti-cancer activity in colorectal cancer in mice [5]. In a previous study the authors reported a bi-phasic effect in human neuroblastoma and glioma for sertraline but not for other SSRIs (e.g., fluoxetine), demonstrating that at low concentrations (<2.5 µM) sertraline caused proliferation and a trophic effect, while higher concentrations induced anti-proliferative and apoptotic effects [10]. 

The present study compared the in vitro and in vivo effects of two SSRIs, sertraline and citalopram, on 4T1 (murine mammary carcinoma) in vitro cell proliferation, and on tumor growth and cancer progression in 4T1 inoculated mice. The in vitro data are consistent with previous reports [10] showing that sertraline alone at concentrations of 20 µM or higher caused a dose-dependent decrease in cell viability while citalopram had no significant effect (Figure 1). Notably, a recent study showed that sertraline may have cytotoxic effects on cultured human peripheral blood lymphocytes, via oxidative stress [25], thus the impact of sertraline on the viability of 4T1 breast cancer cells may be related to a cytotoxic effect rather than to an anti-proliferative effect. 

Under inflammatory conditions (exposure to LPS), only sertraline at concentrations of 2.5 µM or above was able to suppress murine lymphocyte viability. Sertraline alone, at concentrations starting at 5 µM, also inhibited lymphocyte viability (Figure 2). Thus it seems that the beneficial effect of sertraline (at concentrations starting at 2.5 µM) on lymphocytes in an inflammatory environment (LPS stimulation), may have clinical significance.

The in vivo findings indicate that stress may mediate the interaction of sertraline with tumor cells. As previously reported by the authors, there is an immunosuppressive effect of sertraline, expressed in vitro by the inhibition of rodent splenocyte proliferation and the secretion of pro-inflammatory cytokines. The current results are in accordance with the authors’ earlier findings, showing that sertraline but not citalopram induces a concentration-dependent inhibition of splenocyte proliferation under unstimulated conditions as well as following exposure to the mitogen LPS [8,26]. 

Differing effects between the two SSRIs, like the ones found in vitro, were also expected in the in vivo model. The first experiment aimed to evaluate the effects of stress and sertraline therapy on behavior and tumor growth in Balb/c female mice inoculated with 4T1 murine breast cancer cells. Indeed, the results demonstrate that mice exposed to CMS showed higher anxiety compared to vehicle treated mice. Increased anxiety was expressed by decreased activity (frequency, time spent and movement) in the center of an open field. This effect was reversed by sertraline (5 mg/kg/d) treatment (Figure 3), as was the effect on body weight, which was lower in stressed mice and was normalized by sertraline therapy (Figure 4). 

The observations in the current study are in line with those of other studies that reported increased locomotive activity in rodents treated with SSRIs compared to untreated rodents [27]. Yet, at autopsy the tumor weight did not differ among the three cancer-inoculated groups. Thus, contrary to the in vitro data, the in vivo results indicate that at a dose of 5 mg/kg, sertraline was efficacious as an antidepressant but failed to modify tumor growth in stressed mice. 

Following these results, a second experiment was performed using 4T1 inoculated unstressed mice and at a higher dose of sertraline (10 mg/kg/d; IP) to evaluate a possible dose-dependent relationship. The effects of equal doses of sertraline and citalopram were compared. After 20 days, the tumor volume of the sertraline group was larger compared to both the controls and the citalopram group (Figure 5). At autopsy the sertraline treated group tended to have significantly heavier tumors compared to the 4T1 controls, whereas the tumors of the citalopram treated mice were not different from those of the controls, however no significance was obtained in an ANOVA analysis. After a 32-day post inoculation follow up, at autopsy, no significant body weight differences were found among the three cancer group, or the naïve controls.

Additionally, in-line with the data showing a worsening of the tumor conditions when higher doses of sertraline were administered, the survival rate of sertraline treated mice tended to be lower than that of the citalopram treated mice and of the controls, although the differences did not reach statistical significance (Figure 6). In Exp. 2 we used larger doses of SSRIs, hoping that they would prove more effective against tumor progression (at least for sertraline). This, however, made it difficult to compare between Exp. 1 (5 mg/kg) and Exp. 2 (10 mg/kg). 

The current study demonstrates a discrepancy between the in vitro and the in vivo data regarding the effects of sertraline on tumor growth. These data, however, coincide with a clinical report in patients with advanced cancer who received sertraline at 50 mg/d. The sertraline therapy did not improve the symptoms, wellbeing or survival rate of the patients [19]. Moreover, fluoxetine and amitriptyline increased metastases in the DMBA-induced mammary carcinogenesis model [28]. Another study [29] examined the effects of different SSRIs on the outcomes of epithelial ovarian cancer in patients and found that SSRI use was associated with decreased time to disease recurrence. The authors suggested a possible mechanism of increased 5HT in the tumor microenvironment that is involved in mitogenic effects on the ovarian neoplastic cells and is mediated by 5HT2A receptors in the tumor. Contrary to these results, in a cohort of 16,887 breast cancer patients, a concomitant treatment of tamoxifen and antidepressants, including SSRIs, did not result in increased incidence of recurrent disease [30,31]. 

However, these human studies did not investigate the differences in the effects of specific antidepressants. The results of the current study indicate that sertraline and citalopram interact differently with breast tumor cells. Such activity may be also related to the tumor cell type. SSRI therapy in humans has been associated with a reduced risk of colorectal cancer [31], as was also shown in an earlier study by the current study’s authors on colorectal cancer inoculated nude mice [5]. The in vitro suppressive effect of sertraline on tumor cell growth may be related to a mitochondrial cytotoxic effect as reported by Li et al. [32], rather than to an actual anti-proliferative effect. It seems that the effect of an antidepressant on the prognostic parameters of cancer depends on several factors: (1) interaction of the specific SSRI with the tumor cells, (2) the dose used and its potential cytotoxicity, and (3) the specific type of cancer cells. 

Aiming to elucidate the role of the immune system as well, this study determined the ex-vivo responsiveness to the mitogen LPS in splenocytes derived from cancer mice treated with the various regimens. All tumor bearing animals had huge spleens, and following exposure to LPS showed dramatically higher proliferation rates compared to naïve spleens. The 4T1 sertraline group did not differ from the 4T1control group, whereas the 4T1 citalopram-treated group showed a mildly lower responsiveness to LPS (Figure 7). A similar effect was found in the secretion of the pro-inflammatory cytokine TNF-α, which was significantly higher in spleens of the tumor bearing mice than in the spleens of naïve mice, yet no significant differences were found among the cancer groups (Figure 8).

This discrepancy between the in vitro and the ex vivo data suggests that the microenvironment of the tumor in situ and its interaction with the immune system influences the direct effect of the SSRI on the tumor itself. A recent review reported that users of SSRIs and other antidepressants did not experience any notable effect on the risk of colon cancer. Moreover, no clear evidence of either beneficial or harmful association was found between antidepressant treatment and cancer, including colon cancer [33].

Not surprisingly, cancers differ with respect to their effects on cytokine production, metabolism, and transcriptional and translational events throughout the body and within the tumor itself. Thus, the interpretation of a growing repository of similarly obtained data with different types of cancer, "treated" with an inventory of different SSRIs, may yield answers when subjected to pathway analyses. The latter may ultimately guide translational selection of specific SSRIs that are effective for both depressive symptoms and tumor progression in patients with specific types of cancer. Additionally, data subjected to this type of "bioinformatic" scrutiny may help avoid SSRIs that promote the production of pro-inflammatory cytokines, increase the tumorgenicity of the primary cancer, or do little for improving mood. A repository of analyzed data may even suggest repurposed uses of specific antidepressants for unexpected and highly-selective anti-tumor efficacy [14,34,35,36].

In summary, this study showed that sertraline, but not citalopram, inhibits in vitro cancer cell growth. Meanwhile, in vivo sertraline alleviated stress in rodents with grafted cancer cells, but, in contrast to our hypothesis, failed to inhibit in vivo tumor growth. Moreover, in vivo, sertraline, and to a lesser extent, citalopram, demonstrated an accelerated tumor progression effect and tended to lower survival rates (Figure 6). Thus, it appears that not all SSRIs affect cell growth, cell division and cell survival in the same manner and to the same extent. Moreover, data show that a specific SSRI may affect cancer cell growth and survival differently in vivo vs. in vitro, even to the point of opposing effects, as observed with the sertraline in our study.

Notably, no evidence was found in humans of either a beneficial or a harmful association between antidepressant treatment and cancer, including breast cancer and colon cancer [33,37,38]. If, however, the response to SSRIs in humans is indeed similar to that of rodents, then these results indicate that special care has to be taken when prescribing sertraline or citalopram as antidepressants to cancer patients.

## 4. Materials and Methods

### 4.1. Ethics

All treatments and procedures were approved by the Animal Care Committee of Tel-Aviv University and were conducted in accordance with the Guidelines for the Care and Use of Laboratory Animals of the National Institutes of Health, USA (permit number M-11063, given July 2011).

### 4.2. Chemicals and Reagents 

Alamar Blue (Serotec, Kidlington, UK), Fetal Calf Serum [FCS], a combination of Penicillin and Streptomycin, Trypsin, Dulbecco’s Phosphate Buffered Saline [DPBS] and RPMI 1640 (Biological Industries LTD, Kibbutz BeitHa’emk, Israel), Bovine Serum Albumin, Dimethyl Sulfoxide (DMSO) and Neutral Red, Tween 20 and Lipopolysaccharides (LPS; Sigma, St. Louis, MO, USA), Citalopram and Sertraline (Unipharm, Ramat Gan, Israel), splenocyte lysis buffer, composed of 0.01 M, KHCO_3_ and 0.15 M NH_4_Cl in double distilled and filtrated water, Sorenson’s phosphate buffer and Penicillin/Streptomycin, (Biological Industries LTD, Kibbutz BeitHa’emk, Israel), murine tumor necrosis factor (TNF-α; PeproTech, Rocky Hill, NJ, USA).

### 4.3. Cell Line

A murine mammary carcinoma 4T1 cell line (CRL-2539), obtained from ATCC (Rockville, MD, USA), was grown in RPMI with 10% FCS and 1% penicillin/streptomycin (all from Biological Industries LTD, Kibbutz BeitHa’emk, Israel) in 10-cm tissue culture plates. Cells were incubated in a humidified atmosphere of 5% CO_2_ and 95% air at 37 °C.

### 4.4. Primary Splenocyte Cell Culture 

Spleens were removed from naïve female Balb/c mice. The spleens were placed in sterile 100 mm tissue culture dishes (Corning Inc., Corning, NY, USA) containing DPBS with 1% antibiotics (penicillin G 100 U/mL, streptomycin 100 μg/mL). Spleens were homogenized and centrifuged at 320× *g* for 4 min at 25 °C. Red blood cells were lysed with lysis buffer (0.01 M KHCO_3_ and 0.15 M NH_4_Cl) for 40 s and re-centrifuged. The pellets were suspended in RPMI 1640 medium, supplemented with 1% glutamine, 1% antibiotics (penicillin G 100 U/mL, streptomycin 100 μg/mL) and 10% heat-inactivated FCS and incubated in flasks for 24 h at 37 °C, in humidified air containing 5% CO_2_. After 24 h, cells were counted and cultured in the medium 5 × 10^6^ cells/mL in a flat-bottomed 24-well plate (Corning Inc., Corning, NY, USA).

### 4.5. Animals

Ten-week old female Balb/c mice were purchased from Harlan, Jerusalem, Israel. Animals had a seven day acclimatizing period, with five animals housed in each cage, food and water available ad libitum, except when the chronic mild stress (CMS) procedure required deprivation. The standard 12-h light/dark cycle (light from 07:00 to 19:00) was only changed during the CMS. Room temperature was constant at 22 ± 1 °C with 52 ± 2% humidity throughout the experiment. 

Control animals were housed in their cages with no CMS throughout the entire experiment. CMS exposed mice were housed in separate animal rooms at two per cage. Mice received daily intraperitoneal (IP) injections of 5 mg/kg sertraline when assessing CMS effect and 10 mg/kg sertraline when comparing it to citalopram. Both SSRIs were dissolved in saline solution. The control group received a daily IP injection of saline solution. In grafted mice the first drug injection was performed 2 h after injecting them with cancer cells, and the last drug injection was administered 24 h before an animal was sacrificed. 

#### 4.5.1. Chronic Mild Stress 

CMS is an animal model for depression and chronic anxiety, consisting of mild, not traumatic, daily stressors. It includes exposing rodents, for a period of at least two weeks, to mild and unpredictable daily stressors such as a tilted cage, food or water deprivation, paired caging, continuous light, wet bedding, etc. The validity of the model is based on studies showing that CMS induces behavioral changes resembling symptoms of human depression [39,40]. 

In the present study a four to six week CMS regimen, following the protocol described by Ducottet et al. [41], was applied. According to this protocol, the CMS procedure consisted of a variety of unpredictable mild stressors including restraint, forced baths, water and/or food deprivation, pairing with another stressed animal, bedding in wet sawdust, reversing of the light/dark cycle, maintaining constant light or constant darkness. These stressors were administered for periods ranging from 10 min to 24 h over three weeks, according to a daily schedule indicated by Ducottet et al.’s study [41] (Table 1).

#### 4.5.2. Behavioral Anxiety Model 

Open-field tests are commonly used to assess locomotor, exploratory and anxiety-like behavior in laboratory animals [42]. The open-field tasks touch on the conflict between the innate fears of rodents experienced in the central area of a novel or brightly lit open field and their desire to explore new environments. The apparatus consists of four black square arenas with surrounding walls (50 × 50 × 50 cm). Each arena is subdivided into a central zone and a surrounding area (periphery). Experiments were videotaped for 20 min using a video camera. Recordings were analyzed using Ethovision XT software (Noldus, Wageningen, The Netherlands). 

##### T1 Murine Breast Cancer Model

4T1 mammary carcinoma cells in the long phase of growth were harvested and suspended in sterile DPBS at a density of 5 × 10^5^ cells/mL. In order to obtain a solid tumor growth a subcutaneous injection of 0.2 mL of the suspension was injected into the right posterior flank of all animals. Tumor length (L) and width (W) were measured with a caliper, and volume was calculated according to the formula (L × W^2^)/2 [43].

### 4.6. Cell Viability Assay

In all in vitro experiments we used sertraline and citalopram concentrations in the micro-molar range that is usually achieved in humans treated with SSRIs [44]. Thus, the concentration range used in our study is relevant in terms of clinical doses as well. 

4T1 cell viability was assessed using Neutral-Red staining, which is based on the ability of viable cells to incorporate and bind the supravital dye Neutral-Red in the lysosomes [45]. 

Splenocyte viability was assessed using the Alamar Blue method, which is a nontoxic reagent incorporating a redox indicator that changes color in response to mitochondrial metabolic activity [46]. After 48 h, the plate was centrifuged at 1800× *g* for 5 min, and culture supernatants were collected for measurement of cytokine levels. Viability was tested in cells treated in vitro with either sertraline or citalopram alone (both at 0–10 µM) or each with exposure to LPS and compared to controls (untreated cells 5 × 10^6^ cells/mL). The viability values were calculated as a percentage of controls.

### 4.7. In Vivo Experiments

In the in vivo studies, we used doses of 5 mg/kg or 10 mg/kg sertraline or citalopram, as indicated in the two schemes below, since these doses in mice yield blood concentrations in the micro-molar range [47].

#### 4.7.1. Experiment 1—Effects on Anxiety and Tumor Growth of Pre- (14 Days) and Short- (21 Days) or long- (45 Days) Term Treatment with Sertraline (5 mg/kg/d), in Mice Exposed to Chronic Mild Stress

**Scheme 1:**
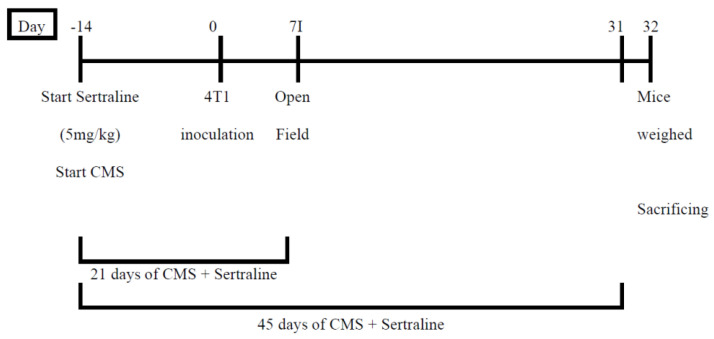
Design of Experiment 1—Effects of pre- (14 days) and short- (21 days) or long- (45 days) term treatment with sertraline (5 mg/kg/d) in mice exposed to chronic mild stress on anxiety (Open Field test) and tumor progression.

Mice were randomly divided into the following 4 groups (5 per group): (1) Intact, (2) 4T1 inoculated controls, (3) 4T1 + CMS and (4) 4T1 + CMS + sertraline (5 mg/kg/d, IP). CMS regimen and sertraline treatment were started two weeks prior to 4T1 cell inoculation, and continued for 31 days after cell inoculation. The reason for the early start of sertraline was intended to test our hypothesis that sertraline acts as a protective/preventive agent. Open Field was conducted on the 7th day after 4T1 cell inoculation. Mice were weighed on day 31 and sacrificed on day 32. Tumors were removed and weighed (Scheme 1).

#### 4.7.2. Experiment 2—Comparison between the Effects on Body Weight, Tumor Growth and Survival of Mice, of Sertraline and Citalopram (10 mg/kg/d Each) Administered after 4T1 Inoculation 

**Scheme 2:**
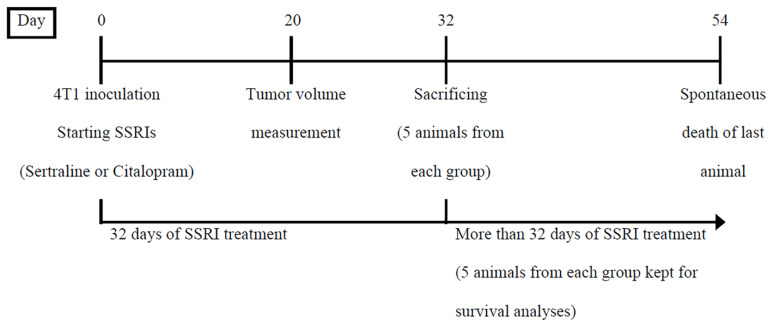
Design of Experiment 2—Comparison between the effects of sertraline and citalopram (10 mg/kg/d each) administered after 4T1 inoculation on body weight, tumor growth and survival of mice.

Mice were randomly divided into the following 4 groups (10 in each group): (1) Control naïve, (2) 4T1 control, (3) 4T1 + sertraline, (4) 4T1 + citalopram. All the mice except for naïve controls were inoculated with 4T1 mammary carcinoma cells. The inoculated mice were treated daily with sertraline (10 mg/kg), citalopram (10 mg/kg) or saline solution (controls). Five animals from each group were sacrificed on day 32. Their bodies and tumors were weighed. The remaining animals continued receiving the SSRI treatment and were kept undisturbed for the second phase of the experiment that investigated survival rates and other parameters (Scheme 2). 

### 4.8. Ex-Vivo TNF-α Cytokine Assay (ELISA)

At the end of the in vivo-exp. 2, mice were sacrificed on day 32. For the next (ex-vivo) experiment, spleens were separated and splenocytes harvested and cultured as described above. Cells were cultured in a 24-well plate at final concentration of 5 × 10^6^ cells/mL under Basal (control) and LPS conditions. Splenocyte suspension was incubated in a total volume of 1 mL per well with 5% CO_2_ at 37 °C. After 48 h, the plate was centrifuged at 1800× *g* for 5 min, and culture supernatants were collected for measurement of cytokine levels using a kit and a protocol purchased from PeproTech Asia (Rehovot, Israel) [26].

This experiment was designed to assess the immune activity of splenocytes, extracted from these mice, as expressed by splenocyte proliferation in response to LPS and TNF-α secretion. These ex-vivo parameters are relevant to anti-tumor activity and are thus related to the topic being investigated in this study.

### 4.9. Statistical Analyses

Analyses of data were performed using One Way Analysis of Variance (ANOVA) or Repeated Measures Analysis of Variance (RM-ANOVA) and the LSD or Sheffe post hoc tests or *t*-test, as appropriate. Statistical significance was set at *p* < 0.05.

## Figures and Tables

**Table 1:** CMS schedule.

	Start of Stress Protocol	Day 1	Day 2	Day 3	Day 4	Day 5	Day 6	Day 7
Week 1	8:00 a.m.	Confinement in a small tube (1 h)	Confinement in a small tube (1 h)	Food restriction (3 h)	Confinement in a small tube (1 h)	Confinement in a small tube (1 h)	Access to an empty bottle (2 h)	Inversion of the light/dark cycle
	2:00 p.m.	Forced bath in water at 32 °C (30 min)	Confinement in a small tube (1 h)	Confinement in a small tube (1 h)	Paired housing in damp sawdust (10 min)	Dark (2 h)	Housing in mildly damp sawdust (24 h)	
	5:00 p.m.		Water and food deprivation (14 h)			Water deprivation (14 h)		
Week 2	8:00 a.m.	Confinement in a small tube (1 h)	Confinement in a small tube (1 h)	Confinement in a small tube (1 h)	Paired housing with food restriction (10 min)	Inversion of light/dark cycle	Inversion of light/dark cycle	Inversion of light/dark cycle
	2:00 p.m.	Forced bath in water at 32 °C (30 min)	Paired housing in damp sawdust (10 min)	Confinement in a small tube (1 h)	Inversion of light/dark cycle			
	5:00 p.m.			Water and food deprivation (14 h)				
Week 3	8:00 a.m.	Confinement in a small tube (1 h)	Food restriction (3 h)	Paired housing in damp sawdust (10 min)	Forced bath in water at 32 °C (30 min)	Access to an empty bottle (2 h)		Inversion of light/dark cycle
	2:00 p.m.	Confinement in a small tube (1 h)	Confinement in a small tube (1 h)	Confinement in a small tube (1 h)	Confinement in a small tube (1 h)	Confinement in a small tube (1 h)	Inversion of light/dark cycle	
	5:00 p.m.	Water and food deprivation (14 h)			Water deprivation (14 h)	Housing in mildly damp sawdust (24 h)		

## Data Availability

Below are suggested Data Availability Statements: Data is contained within the article. All the data presented are included in the study.
